# Breast cancer histopathological images recognition based on two-stage nuclei segmentation strategy

**DOI:** 10.1371/journal.pone.0266973

**Published:** 2022-04-28

**Authors:** Hongping Hu, Shichang Qiao, Yan Hao, Yanping Bai, Rong Cheng, Wendong Zhang, Guojun Zhang

**Affiliations:** 1 School of Science, North University of China, Taiyuan, China; 2 School of Information and Communication Engineering, North University of China, Taiyuan, China; 3 School of Instrument and Electronics, State Key Laboratory of Dynamic Testing Technology, North University of China, Taiyuan, China; PDPM IIITDM: PDPM Indian Institute of Information Technology Design and Manufacturing Jabalpur, INDIA

## Abstract

Pathological examination is the gold standard for breast cancer diagnosis. The recognition of histopathological images of breast cancer has attracted a lot of attention in the field of medical image processing. In this paper, on the base of the Bioimaging 2015 dataset, a two-stage nuclei segmentation strategy, that is, a method of watershed segmentation based on histopathological images after stain separation, is proposed to make the dataset recognized to be the carcinoma and non-carcinoma recognition. Firstly, stain separation is performed on breast cancer histopathological images. Then the marker-based watershed segmentation method is used for images obtained from stain separation to achieve the nuclei segmentation target. Next, the completed local binary pattern is used to extract texture features from the nuclei regions (images after nuclei segmentation), and color features were extracted by using the color auto-correlation method on the stain-separated images. Finally, the two kinds of features were fused and the support vector machine was used for carcinoma and non-carcinoma recognition. The experimental results show that the two-stage nuclei segmentation strategy proposed in this paper has significant advantages in the recognition of carcinoma and non-carcinoma on breast cancer histopathological images, and the recognition accuracy arrives at 91.67%. The proposed method is also applied to the ICIAR 2018 dataset to realize the automatic recognition of carcinoma and non-carcinoma, and the recognition accuracy arrives at 92.50%.

## 1 Introduction

In recent years, the incidence and mortality of global cancer have been rising continuously, which seriously threatens human life and health. Breast cancer is one of the cancers with the highest mortality for females in the world [1]. One of the most obvious changes in the latest global cancer data in 2020 is the rapid increase in the number of new cases of breast cancer, which has replaced lung cancer to be the world’s leading cancer [2]. Breast cancer pathological examination is considered to be the gold standard for breast cancer diagnosis. The recognition of histopathological images of breast cancer has attracted a lot of attention in the field of medical image processing. Nowadays the breast cancer diagnosis mainly depends on the priori knowledge and diagnostic experience of pathologists. During the diagnosis process, the essence of abnormal tissues cannot be recognized sometimes, and even false detection and missed detection may occur. Therefore, researchers assist doctors in processing and analyzing medical images through imaging, medical images processing technology and computer analysis and calculation, that is, computer aided diagnosis (CAD) system.

With the advancement of CAD technology, machine learning has been widely used in the diagnosis of breast cancer [3–6]. Effective feature extraction is the key to histopathological images recognition, but the realization of the automatic recognition of breast cancer histopathological images is a challenging task to due to the characteristics of histopathological images. At present, the traditional methods used for breast cancer histopathological images recognition mainly consist of the artificial feature extraction methods and deep learning methods [7–10].

The traditional artificial feature extraction methods require manually designing the region of interest in the images, and the features are extracted and then the extracted features are needed to be selected. In [11], a breast cancer histopathological images dataset called BreaKHis was proposed by Spanhol et al. for preforming the benign and malignant classification of tumors by six different extracted features: completed local binary pattern(CLBP), gray level co-occurrence matrix (GLCM), local binary pattern (LBP), local phase quantization (LPQ), parameter-free threshold adjacency statistics (PFTAS) and one keypoint descriptor named Oriented FAST and Rotated BRIEF (ORB) features, and four kinds of different classifiers: 1-nearest neighbor (1-NN), quadratic linear analysis (QDA), random forests (RF) and support vector machine (SVM). In [12], Belsare et al. firstly used the spatial color texture image segmentation method to segment the images, then extracted the features: GLCM, graph running length matrix and Euler number, and used linear discriminant analysis (LDA), to perform the classification of the breast cancer histopathological images. Reis et al. combined multi-scale basic image features and LBP features with random decision trees to make the maturity of the stroma in the breast tissue be classified [13]. Chan et al. applied fractal dimension features to breast cancer detection [14]. Hao et al. extracted three-channel features of 10 feature descriptors on the BreaKHis dataset to classify breast cancer histopathological images [15].

Deep learning methods have also been widely used in breast cancer histopathological images recognition. Araújo et al. used Convolutional Neural Network (CNN) and CNN combined with SVM for the binary classification based on the Bioimaging 2015 dataset [16]. Wang et al. classified the ICIAR 2018 dataset into four categories through the VGG16 network and the transfer learning [17]. Spanhol et al. also adopted AlexNet for breast cancer classification based on BreaKHis and achieved better results than the machine learning model trained with hand-extracted texture descriptors [18]. Saini et al. firstly used deep convolution generation adversarial network to augment the data of benign samples, and then used the improved VGG16 to extract the features of different pooling layers, and SVM was used to classify breast cancer histopathological images [19]. Roy et al. used convolutional neural networks to automatically classify breast cancer histopathological images [20]. Brancati et al. fine-tuned ResNet and tested the model on the ICIAR 2018 and Bioimaging 2015 datasets [21]. Rakhlin et al. used several deep neural network models and gradient enhanced tree classifiers to carry out classification research on ICIAR 2018 dataset [22]. Kassani et al. proposed a method of automatic binary classification of breast cancer histopathological images based on integrated deep learning [23]. Alom et al. proposed the Inception Recurrent Residual Convolutional Neural Network (IRRCNN) model and applied it to the classification of the BreaKHis and Bioimaging 2015 datasets [24].

Besides the commonly used artificial feature extraction methods and deep learning methods, many scholars have also applied multi-instance learning and sparse representation methods to recognize the breast cancer histopathological images. Sudharshan et al. used a multi-instance learning method to classify the BreaKHis dataset into benign and malignant categories [25]. A new multi-channel histopathological image simultaneous sparse model was proposed by Srinivas et al. and was applied to solve a new optimization problem based on simultaneous sparseness for performing breast cancer histopathological images classification [26]. Li et al. proposed the combination of the discriminative feature learning and the multi-channel joint sparse representation based on mutual information for classifying benign and malignant tumors at 40× magnification on the BreaKHis dataset [27]. In addition, the distribution, size and morphology, and aggregation density of cell nuclei are the important information of breast cancer histopathological images. Therefore, the researches on the cell nuclei segmentation and the cell morphology are the significant importance for breast cancer histopathological images recognition. Kumar et al. proposed a framework for automatic detection and classification of cancer from microscopic biopsy images, which includes cell segmentation, feature extraction, and classification [28]. Kowal et al. used four different clustering methods and the adaptive gray thresholds to segment cell nuclei, and then extracted 42 morphological, topological and texture features for breast cancer benign and malignant classification [29]. Zheng et al. used the blob detection method to detect the nucleus whose location was determined by use of the local maximum, and used the sparse autoencoding to extract features of the nucleus slice for the recognition of benign and malignant breast tumors [30]. Anuranjeeta et al. extracted the shape and morphological features of cells for breast cancer classification and recognition [31]. Pang et al. trained CNN using gradient descent technology to solve the problem of cell nuclei segmentation for histopathological images [32].

**Fig 1:**
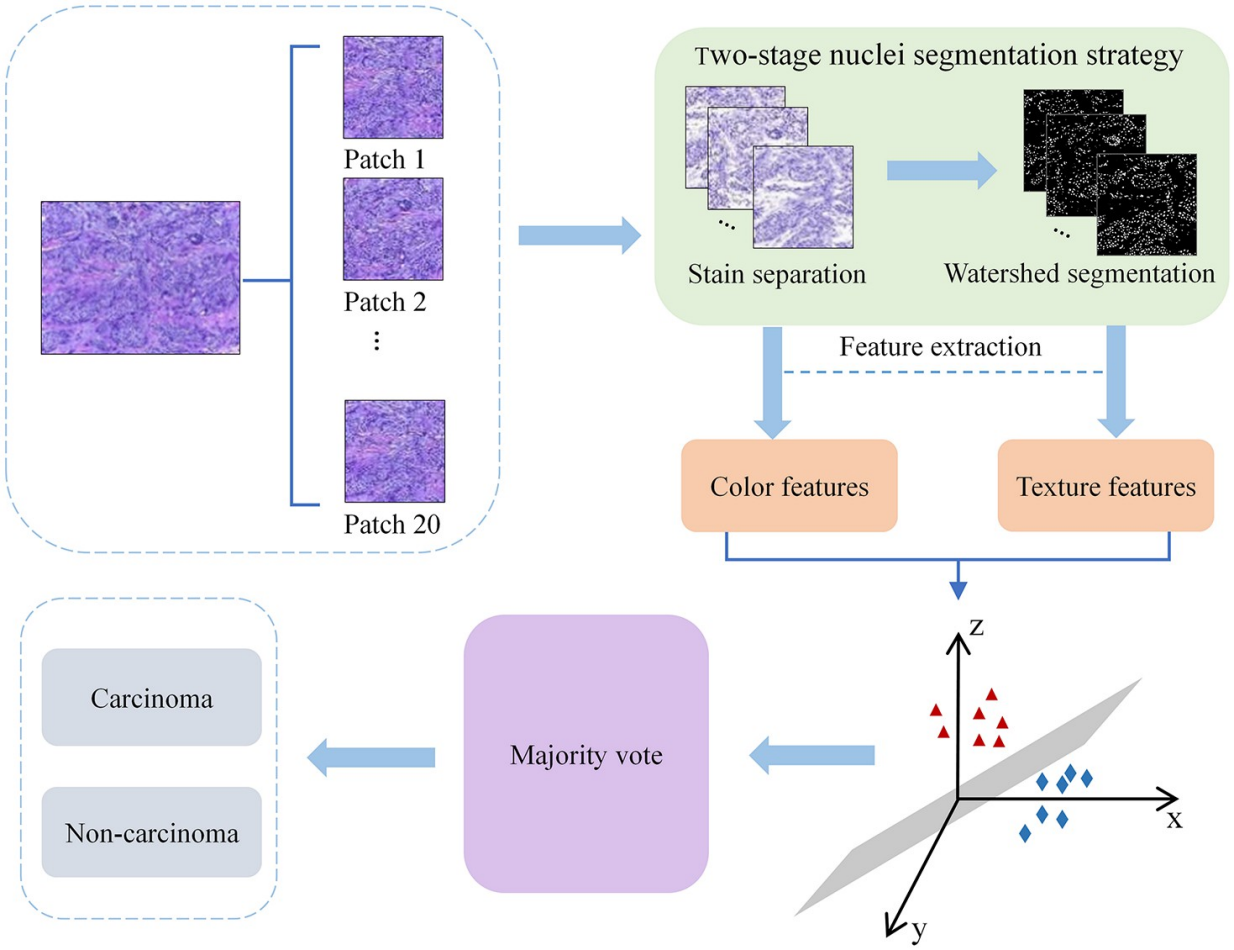
The framework of breast cancer histopathological images recognition based on the two-stage nuclei segmentation strategy proposed in this paper.

For the problems of under-segmentation and over-segmentation in the process of histopathological images segmentation, a two-stage nuclei segmentation strategy, that is, a method of watershed segmentation based on histopathological images after stain separation, is proposed on the base of the Bioimaging 2015 dataset in this paper to make the dataset recognized to be the carcinoma and non-carcinoma recognition. Firstly, stain separation is performed on breast cancer histopathological images. Then the marker-based watershed segmentation method is used for images obtained from stain separation to achieve the nuclei segmentation target. Next, the completed local binary pattern was used to extract texture features from the nuclei regions (images after nuclei segmentation), and color features were extracted by using the color auto-correlation method on the stain-separated images. Finally, the two kinds of features were fused and the support vector machine was used for carcinoma and non-carcinoma recognition. The experimental results show that the two-stage nuclei segmentation strategy proposed in this paper has significant advantages in the recognition of carcinoma and non-carcinoma on breast cancer histopathological images, and the recognition accuracy arrives at 91.67%. The proposed method is also applied to the ICIAR 2018 dataset to realize the automatic recognition of carcinoma and non-carcinoma, and the recognition accuracy arrives at 92.50%. Fig 1 shows the framework of breast cancer histopathological images recognition based on the two-stage nuclei segmentation strategy proposed in this paper.

In this paper, an effective automatic computer-aided diagnosis technique is proposed for the segmentation and recognition of breast cancer histopathological images. This work makes the significant contributions to the realization of an interactive system for nuclei segmentation and cancer recognition, as follows:

1. A two-stage nuclei segmentation strategy is proposed for nuclei segmentation of histopathology images. It is a challenging task to achieve nuclei segmentation in histopathology images with similar foreground and complex background. The proposed method not only effectively avoids the under-segmentation and over-segmentation problems, but also provides good cancer detection performance with less algorithm complexity and faster running speed.
2. Based on the two-stage nuclei segmentation strategy, a breast cancer histopathology image recognition model for cancer detection is proposed. This model is performed on two different modes: patches-wise and image-wise. Cancer can be effectively identified by extracting low-dimensional features based on nuclei segmentation, and it has good cancer recognition performance on two kinds of different datasets, which has wide applicability and can replace deep learning methods to some extent. The method can provide a diagnostic review technique to reduce human error for pathologists.

The rest of the paper is organized as follows: in Section 2, a two-stage nuclei segmentation strategy was proposed. In Section 3, the feature extraction methods were introduced in detail. Section 4 is the experimental results and Section 5 is the discussion and conclusion.

## 2 The proposed two-stage nuclei segmentation strategy

Due to the characteristics of histopathological image, it is a challenging task to perform the automatic classification of the histopathological images of breast cancer. The overlapping of cells, uneven color distribution and subtle differences between images have brought the great difficulties to the classification of breast cancer histopathological images [33]. The effective and sufficient nuclei segmentation of histopathological images can improve the classification performance. However, in histopathological images, the diversity, the density and the overlap of nuclei pose the great challenges for the nuclei segmentation task of histopathological images [34]. In order to fully segment the nuclei, get more effective features, and prevent the under-segmentation and the over-segmentation, a two-stage nuclei segmentation strategy is proposed in this paper: stain separation is firstly conducted on the breast cancer histopathological images to obtain the foreground images, then the nuclei are segmented by the watershed segmentation method on the image after stain separation, thus the obtain images have a better degree of segmentation and more effective information.

### 2.1 Stain separation

The stain separations of histopathological images are helpful for pathologists and CAD system. Separation techniques used for natural images may cause changes in the structural characteristics of stained tissues in histopathological images and produce undesirable color distortions. The method commonly used in Hematoxylin and Eosin (H&E) image stain separation is realized by converting the RGB space to the optical density. Since the stain separation is an estimation of the density map of each stain, the relationship between the RGB color and the stain density of each pixel needs to be considered: the stained tissue will weaken the light in a certain spectrum according to the type and the amount of the absorbed stain. In this paper, the stain separation method based on the Sparse Non-negative Matrix Factorization (SNMF) framework proposed in [35] was used for breast cancer histopathological images stain separation.

Let *I* ∈ *R*^*m*×*n*^ be the matrix of the RGB intensities, where *m* = 3 is the number of the RGB channels, and *n* is the total number of image pixels. And let *I*_0_ be the illuminating light intensity on the sample (usually 255 for 8 bit images). Then the relative optical density *V* can be expressed to be as follows [36]:

$$V=\text{log}\frac{I_{0}}{I}.$$ (1)

Let *V* = *WH*, *W* ∈ *R*^*m*×*r*^ be the stain color appearance matrix whose columns represent the color basis of each stain such that *r* is the number of stains, and *H* ∈ *R*^*r*×*n*^ be the stain density maps, whose rows represent the concentration of each stain. Therefore, for an given observation matrix *V*, the stain color appearance matrix *W* and stain density map matrix *H* need to be obtained from solving the following problem:

$$\underset{W,H}{\text{min}}\frac{1}{2}{\left\|V-WH\right\|}_{F}^{2},W,H\geq 0.$$ (2)

Since this problem (2) is a non-convex optimization problem where the local optimum is obtained instead of the global optimum, an undesirable coloring vector is obtained. Therefore, Vahadane et al. [35] proposed a sparse non-negative matrix factorization (SNMF) framework where a sparseness constraint is added into Eq (2) and thus the Eq (2) is become to be as follows:

$$\underset{W,H}{\text{min}}\;\frac{1}{2}{\left\|V-WH\right\|}_{F}^{2}+\lambda \sum_{j=1}^{r}\left\|H(j,:)\right\|{\;}_{1},W,H\geq 0,{\left\|W(:,j)\right\|}_{2}^{2}=1,$$ (3)

**Fig 2:**
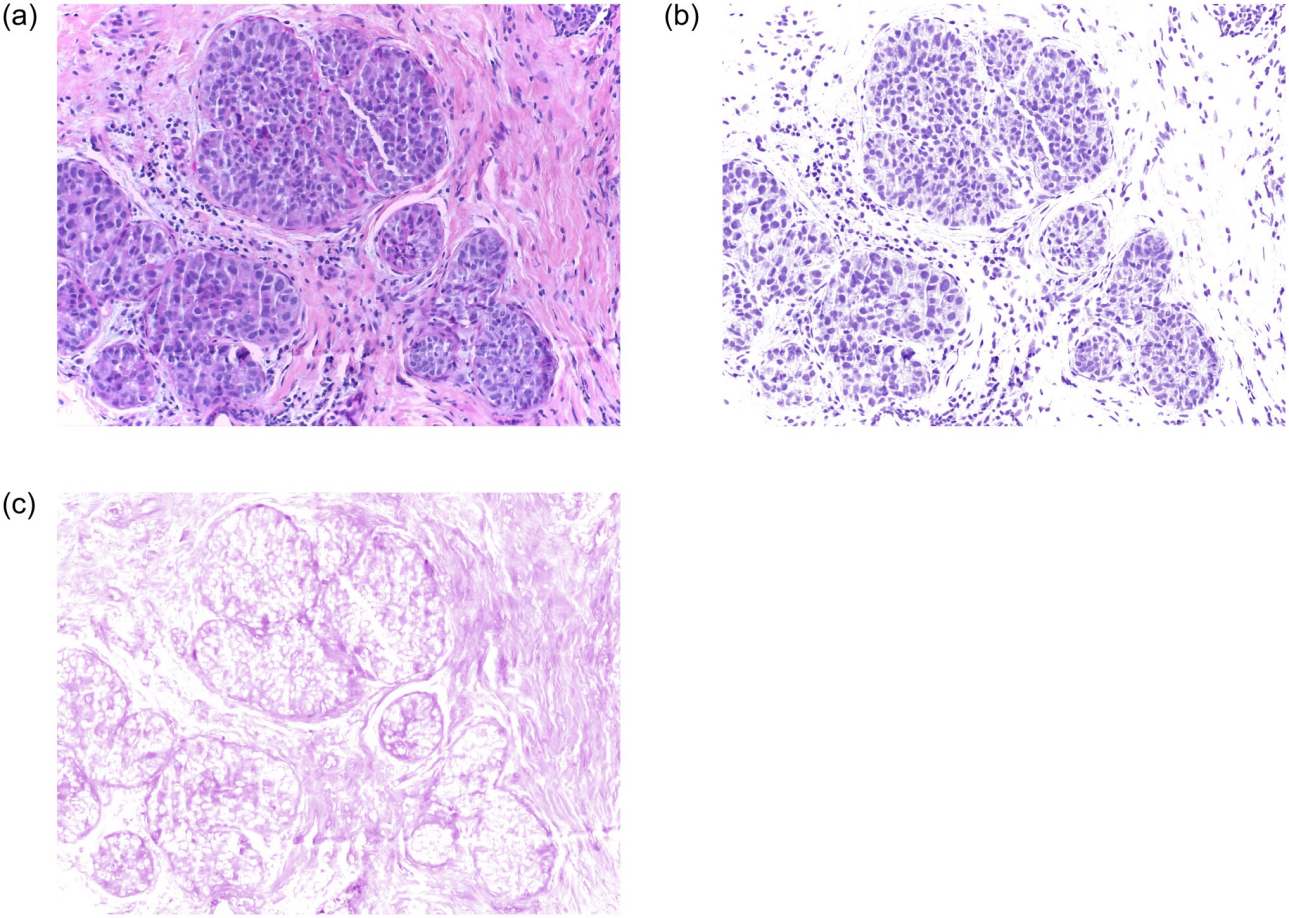
Stain separation results of breast cancer histopathological images. (a) Original image, (b) Fore ground image of stain separation, (c) Back ground image of stain separation.

Where ∥·∥_*F*_ denotes the F-norm of a matrix, and *λ* = 0.2 is the sparsity and regularization parameter, and *j* indicates the type of stains (*j* = 1, 2, …, *r*). For the H&E images, *r* = 2. The LARS-LASSO algorithm [37] can be applied to solve the Eq (3), then *W* and *H* are obtained, and then the stain separations of H&E images are preformed. Fig 2 shows the stain separation results of the images on the Bioimaging 2015 dataset using the above method: stain separation.

### 2.2 Nuclei segmentation

Nuclei segmentation is a basic but challenging task in the histopathological image analysis. Compared with the segmentation of independent nucleus, the segmentation of overlapping and adherent nuclei is a key of histopathological image segmentation in recent years. The morphological changes of the nuclei are considered to be the important information for many diseases. The distribution, size and density of nuclei reflect the pathological changes of breast cancer, which are the important basis for judging carcinoma and non-carcinoma. The common segmentation methods consist of the threshold segmentation, the edge detection, the active contour, the k-means clustering segmentation and the watershed segmentation. In this paper, the watershed segmentation is used to segment the nuclei of breast cancer histopathological images obtained from stain separation.

Watershed algorithm is an image segmentation algorithm based on mathematical morphology. The image is regarded to be a topological landform, where each pixel represents the altitude of the point, each local minimum and its affected area are called catchment basin, and the boundary forms a watershed. The watershed segmentation algorithm is applied to extract the pixels based on the similarity between the pixels. For the extraction and segmentation of cell nuclei, each pixel value in the histopathological images is regarded to be the altitude of a pixel the in the watershed algorithm. The commonly watershed algorithms include watershed segmentation based on distance transformation, gradient-based watershed segmentation, and marker-based watershed segmentation.

Since over-segmentation is prone to exist in the watershed algorithm, the noise or other interference factors on the images will also affect the watershed segmentation for histopathological images. In order to solve the over-segmentation problem, the marker-based watershed segmentation algorithm is selected in this paper. The marker-based watershed segmentation algorithm is applied to perform the watershed segmentation on the gradient image of the original image rather than indirectly on the original image, which ensure the integrity of the edge information of the target object as far as possible and avoid over-segmentation of histopathological images. Therefore, in order to reduce the influence of noise and other interference factors on nuclei segmentation in the breast cancer histopathological images, the marker-based watershed segmentation is applied into the breast cancer histopathological images obtained from the stain separation in this paper.

### 2.3 Two-stage nuclei segmentation strategy based on stain separation and watershed algorithm

The detection of visually salient image regions [38] is very useful for image segmentation. Therefore, the Frequency-tuned salient region detection method is applied into the original marker-based watershed segmentation algorithm for the sake of the segmentation performance improvement. The method exploits feature of color and luminance and outputs full resolution saliency maps with well-defined boundaries of salient objects. With the sensation of image segmentation, the noise in the corners of the image is removed before segmentation.

**Fig 3:**
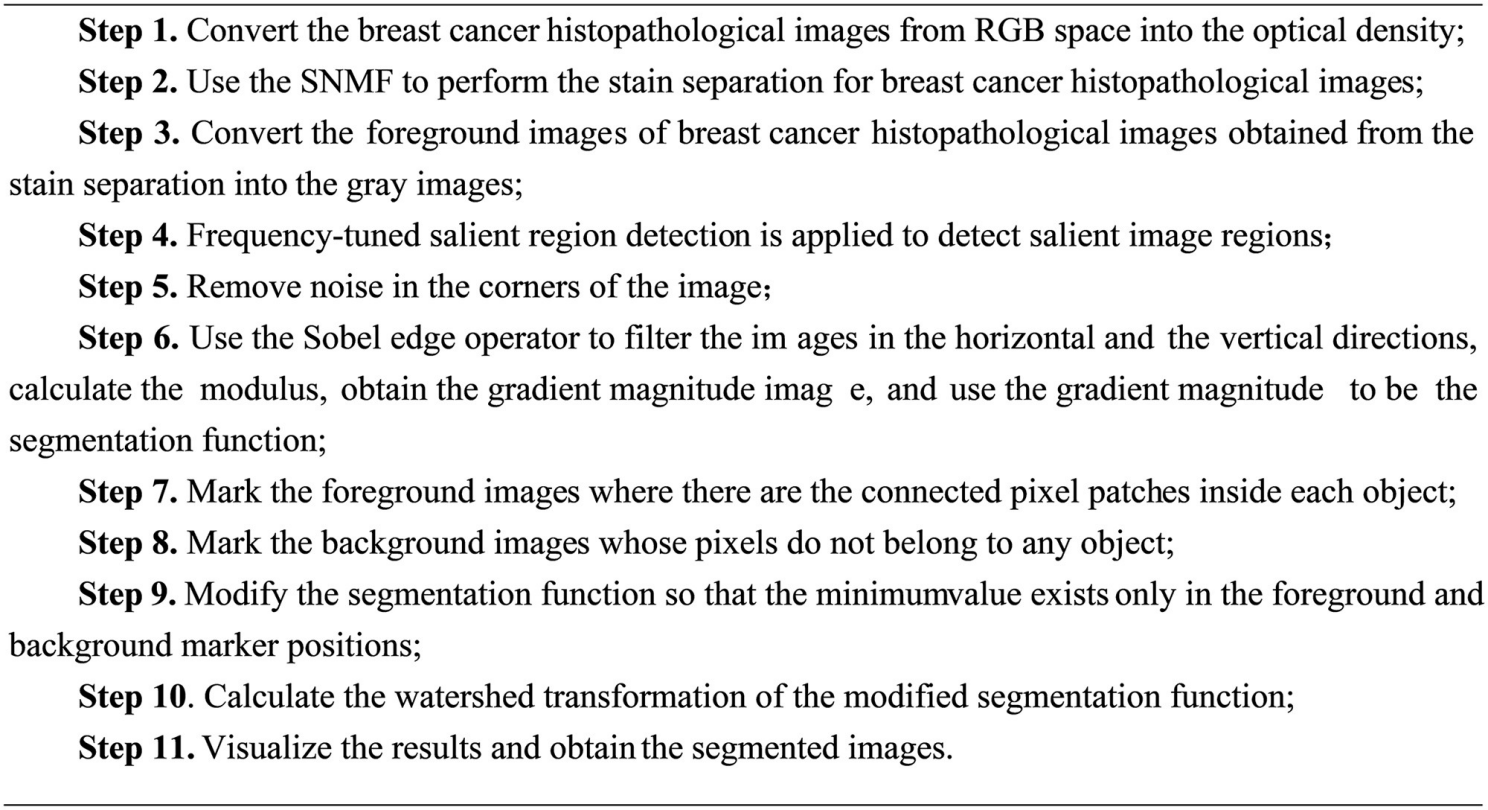
The steps of the two-stage segmentation strategy.

**Fig 4:**
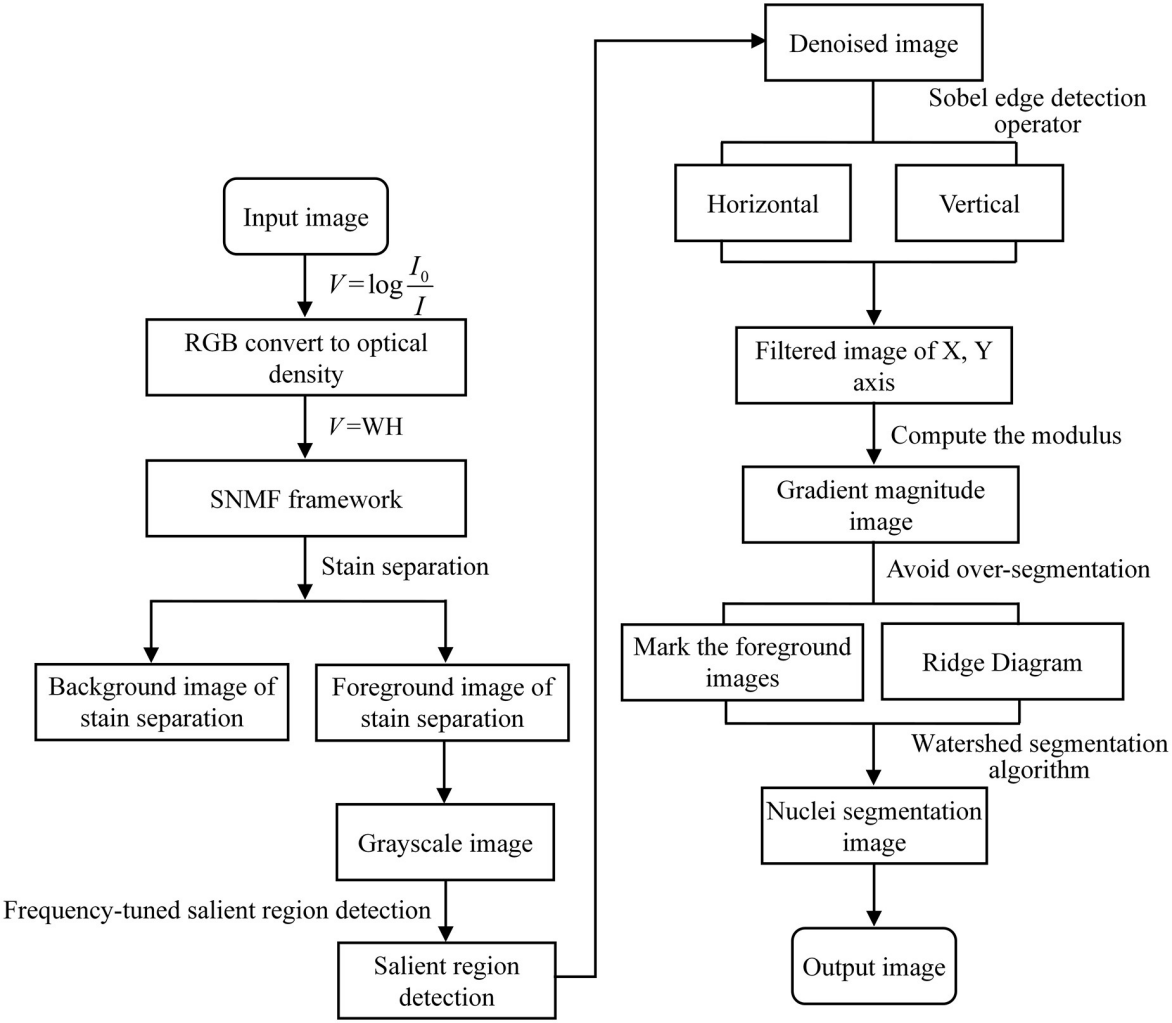
Flowchart of the proposed two-stage nuclei segmentation method.

**Fig 5:**
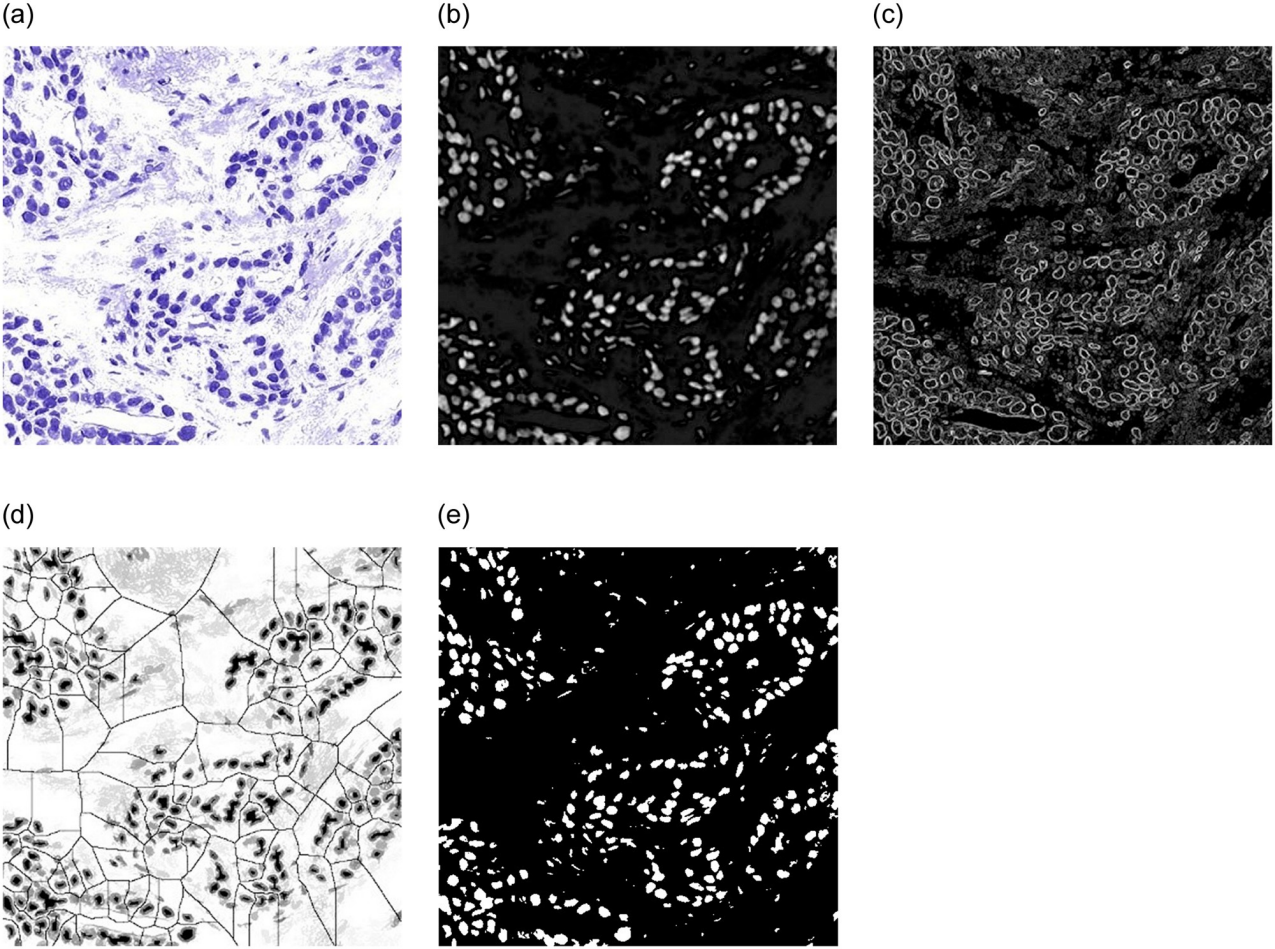
Visualization of two-stage nuclei segmentation. (a) Stain separation image, (b) Salient Region Detection, (c) Gradient amplitude image, (d) Marked image, (e) Nuclei segmentation image.

The steps of the two-stage segmentation strategy based on the stain separation and the watershed algorithm proposed in this paper are as shown in Fig 3. And Fig 4 is the flowchart of the proposed two-stage nuclei segmentation method. Fig 5 shows salient region detection, the gradient amplitude image, the marked image, and the final segmentation results obtained by applying the proposed two-stage nuclei segmentation strategy into the breast cancer histopathological images.

**Fig 6:**
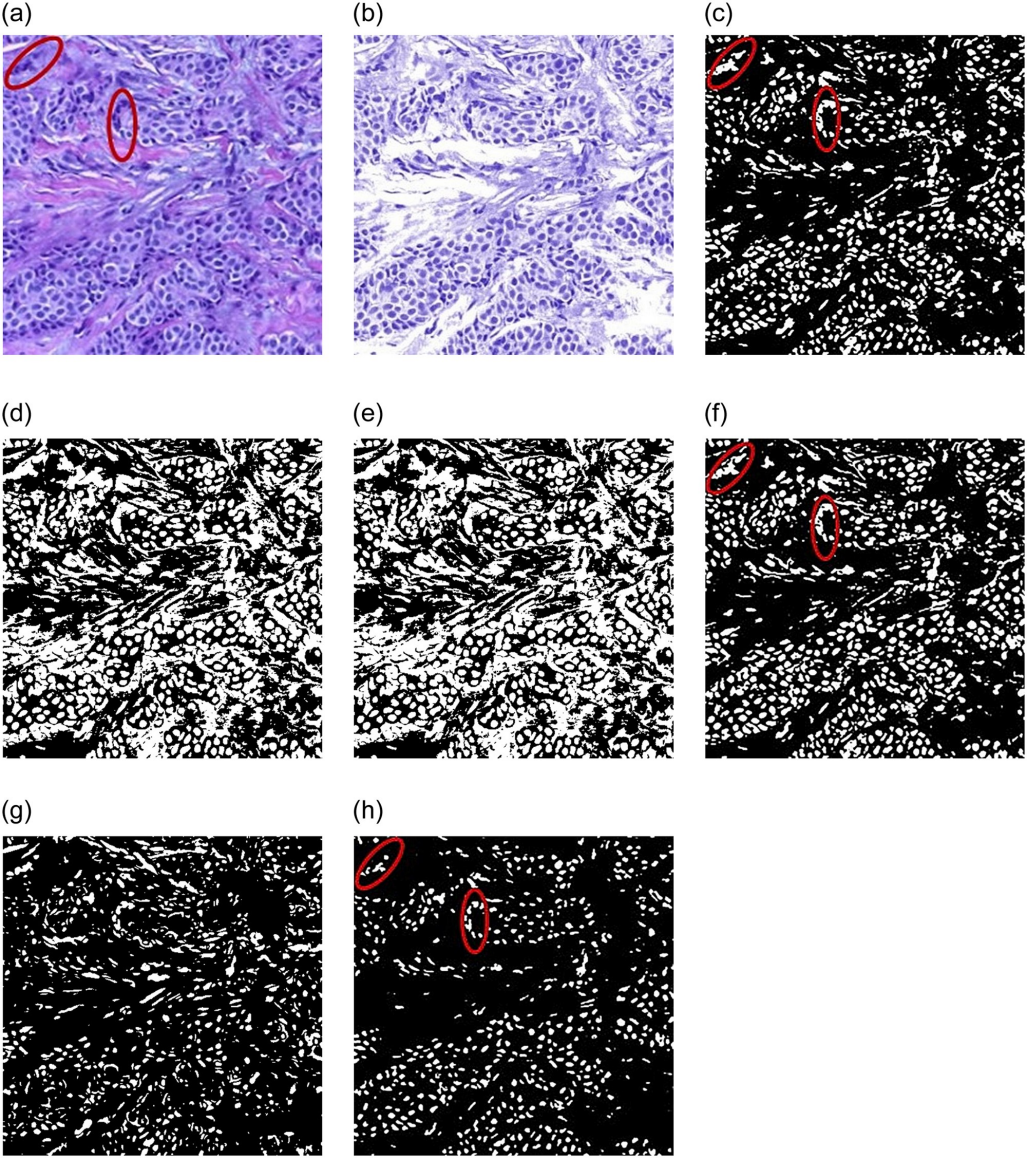
Results of different segmentation methods. (a) Original image, (b) Stain separation image, (c) k-means clustering segmentation, (d) Ostu threshold segmentation, (e) Iterative threshold segmentation, (f) Minimum error threshold segmentation method, (g) Watershed segmentation directly used for the original image, (h) Two-stage segmentation strategy.

The proposed two-stage segmentation strategy based on stain separation and watershed algorithm in this paper is compared with four different segmentation methods: k-means clustering segmentation, Ostu threshold segmentation (maximum between-cluster variance method), minimum error threshold segmentation, and iterative threshold segmentation. In addition, the watershed segmentation directly used for the original image is compared with the proposed segmentation method. The comparing results on breast cancer histopathological images are shown in Fig 6. Fig 6a is the original image, where the red marked area is the nuclei with adhesion and overlapping, and Fig 6b is the fore ground image obtained from stain separation. By comparison and observation from Fig 6, the Ostu threshold segmentation and the iterative threshold segmentation have the worst performance, but fail to accurately segment the nucleus, as shown in Fig 6d and 6e, respectively; the k-means clustering segmentation and the minimum error threshold segmentation method can accurately segment the nuclei, but for some nuclei with overlapping and adhesion in histopathological images, the edges cannot be accurately segmented, and there is still adhesion and overlapping in the segmented image, as marked to be the red cycles in Fig 6c and 6f, respectively; the proposed two-stage segmentation strategy can not only completely and fully segment the nucleus, but also performs well on the nuclei that are adhered and overlapped, as marked to be the red cycles in Fig 6h. The image obtained by the watershed segmentation directly used for the original image has more noise and over-segmentation phenomenon and the segmentation effect is far inferior to the proposed segmentation method, shown in Fig 6g.

### 2.4 Computational complexity

The complexity of the two-stage nuclei segmentation strategy method mainly depends on the implementation processes of the stain separation and the marker-based watershed segmentation algorithm. The algorithm complexities of the stain separation and the segmentation process are analyzed respectively.

#### 2.4.1 The complexity of stain separation

As already introduced in Section 2.1, the SNMF framework is used in the process of stain separation, and sparse constraints is added to obtain a LASSO problem, which is solved by the LARS-LASSO algorithm. Therefore, the complexity of the stain separation process mainly depends on the calculation of the LARS-LASSO algorithm. LASSO is a constrained version of Ordinary Least Squares (OLS). Let *x*_1_, *x*_2_, …, *x*_*m*_ be *n*-dimensional vectors, *A* ∈ *R*^*n*×*m*^, and *y* be an *n*-dimensional vector. Then the model of lasso is as follows:

$$\underset{x}{\text{min}}\frac{1}{2}{\left\|y-Ax\right\|}_{2}^{2}+\lambda {\left\|x\right\|}_{1}.$$ (4)

In response to this problem, LARS algorithm proposed by Efron [37] is a more prudent method of single variable selection, whose complexity is equivalent to that of OLS. The entire sequence of steps in the LARS algorithm with *m* < *n* variables requires *O*(*m*^3^ + *nm*^2^) computations. For the lasso, costing at most *O*(*m*^2^) operations per downdate. Therefore, the complexity of stain separation is *O*(*m*^3^ + (*n* + 1)*m*^2^).

#### 2.4.2 The complexity of the segmentation process

The Frequency-tuned salient region detection method is applied into the original marker-based watershed segmentation algorithm for the sake of detecting salient image regions [38]. The computational complexity of this method is *O*(*N*), where *N* is the scale of the algorithm. In the segmentation process, with the corner denoising operation performed, computational complexity of the overall segmentation process proposed in this paper is *O*(*N*^2^).

In addition, in order to show the time complexity more clearly, we counted the running time of 10 breast cancer histopathological images in the process of stain separation and segmentation respectively, and the image size is 512×512. Completed 10 experiments to obtain the average time, and obtained the processing time of each image in the process of stain separation and segmentation. The results show that the stain separation and segmentation process of each image takes about 10.99s and 0.89s, respectively. Therefore, the method proposed in this paper is a simple and feasible method that does not depend on hardware equipment.

## 3 Feature extraction

In the image recognition, a lot of redundant information exists in the original image, which seriously affects the classification accuracy of the image. It is crucial for image recognition to choose an appropriate feature extraction method. The effective information is extracted, and the dimension of the feature is reduced at the same time, which avoids the disaster of dimension. The common methods of the extracting texture features include gray-level co-occurrence matrix, Tamura feature, wavelet transform, Gabor feature, Completed Local Binary Pattern (CLBP), etc. [39–42]. The common methods of the extracting color features include color histograms, color moments, and color auto-correlogram. In this paper, the CLBP method is used to extract the texture features of the breast cancer histopathological images obtained from nuclei segmentation, and the color auto-correlogram is used to extract the color features of the fore ground image of the breast cancer histopathological images obtained from stain separation.

### 3.1 The central gray of Completed Local Binary Pattern (CLBP)

CLBP is a variant of Local Binary Pattern (LBP). The local area of the CLBP operator is represented by its center pixel and the sign-magnitude transformation of local difference. After global thresholding, the central pixel is encoded by binary string, thus CLBP is called to be the central gray of complete local binary pattern (*CLBP*_*C*). Meantime, the sign-magnitude transformation of local difference is decomposed into two complementary structural components: difference sign CLBP-Sign (*CLBP*_*S*) and difference magnitude CLBP-Magnitude (*CLBP*_*M*). For a pixel (*x*_*c*_, *y*_*c*_) in the image, the components *CLBP*_*C*, *CLBP*_*S* and *CLBP*_*M* are to be as follows:

$$\left\{\begin{array}{l} CLBP\_C_{P,R}\left(x_{c},y_{c}\right)=s\left(g_{c}-g_{N}\right)\\ CLBP\_S_{P,R}\left(x_{c},y_{c}\right)=\sum_{p=0}^{P-1}s(g_{p}-g_{c})2^{p}\;s(x)=\left\{\begin{array}{l} 1,x\geq 0\\ 0,x<0 \end{array}\right.\\ CLBP\_M_{P,R}\left(x_{c},y_{c}\right)=\sum_{p=0}^{P-1}s(D_{p}-D_{c})2^{p} \end{array}\right.,$$ (5)

where *P* is the number of sampling points in the neighborhood of the center pixel, *R* is the radius of the neighborhood, *g*_*c*_ is the gray value of the center pixel, $g_{N}=\frac{1}{N}\sum_{n=0}^{N-1}g_{n}$ represents the mean gray value about *g*_*c*_ when the center point is constantly moving, *N* is the number of windows, *g*_*p*_ is the gray value of the pixel adjacent to the center pixel, *D*_*p*_= |*g*_*p*_ − *g*_*c*_|, and $D_{c}=\frac{1}{P}\sum_{p=0}^{P-1}\left|g_{p}-g_{c}\right|$ represents the mean magnitude.

In Eq (5), *CLBP*_*S*_*P*,*R*_(*x*_*c*_, *y*_*c*_) is equivalent to the traditional LBP operator, which describes the difference sign feature of the local window; *CLBP*_*M*_*P*,*R*_(*x*_*c*_, *y*_*c*_) describes the difference magnitude characteristics of the local window; and *CLBP*_*C*_*P*,*R*_(*x*_*c*_, *y*_*c*_) is the gray level information reflected by the pixel at the center.

### 3.2 Color auto-correlogram

The color features are the basic visual features of color images. Compared with other visual features, they are less dependent on the direction, size, and viewing angle of the image, and are related to the objects or scenes contained in the image. The color histogram describes the proportion of different colors in the entire image, but cannot describe the objects in the image. The color moment generally has only 9 components (3 color components, 3 low-order moments on each component), and the feature dimension is small, which makes it difficult to completely describe the color information of the image. The color auto-correlogram is obtained from the color correlogram. The color correlogram can not only reflect the proportion of the number of pixels of a certain color in the entire image in an image, but also reflect the spatial correlation between different color pairs [43]. For image *I*, let *I*_*c*(*i*)_ be the all pixels of color *c*(*i*), then the color correlogram can be written as:

$$r_{c(i),c(j)}^{\left(k\right)}=P_{r}[|p_{1}-p_{2}|=k]\;p_{1}\in I_{c(i)},p_{2}\in I_{c(j)},$$ (6)

Where |*p*_1_ − *p*_2_| represents the distance between *p*_1_ and *p*_2_, *P*_*r*_ is the calculation of probability. That is, the color correlogram can be regarded as a table indexed by a color pair <*i*, *j*>, the *k*-th component of <*i*, *j*> represents the probability that the distance between the pixel with color *c*(*i*) and the pixel with color *c*(*j*) is equal to *k*. If the correlation between any colors in the image is considered, the color correlogram of the image will be very complicated and huge. If only considers the spatial relationship between pixels with the same color is only considered, the color correlogram is to be the color auto-correlogram.

Due to the limitations of color histograms and color moments, color auto-correlogram is used to describe the color features of breast cancer histopathological images in this paper. In this paper, CLBP is applied to extract the texture features of the image obtained from nuclei segmentation. Let *P* = 8, *R* = 1, then, get the 118-dimensional feature vector. The method of color auto-correlogram is used to extract the 128-dimensional feature vector as the color feature of the breast cancer histopathological image obtained from stain separation. The above two features are cascaded and input into SVM for breast cancer histopathological images recognition.

## 4 Experimental results

### 4.1 Dataset

**Fig 7:**
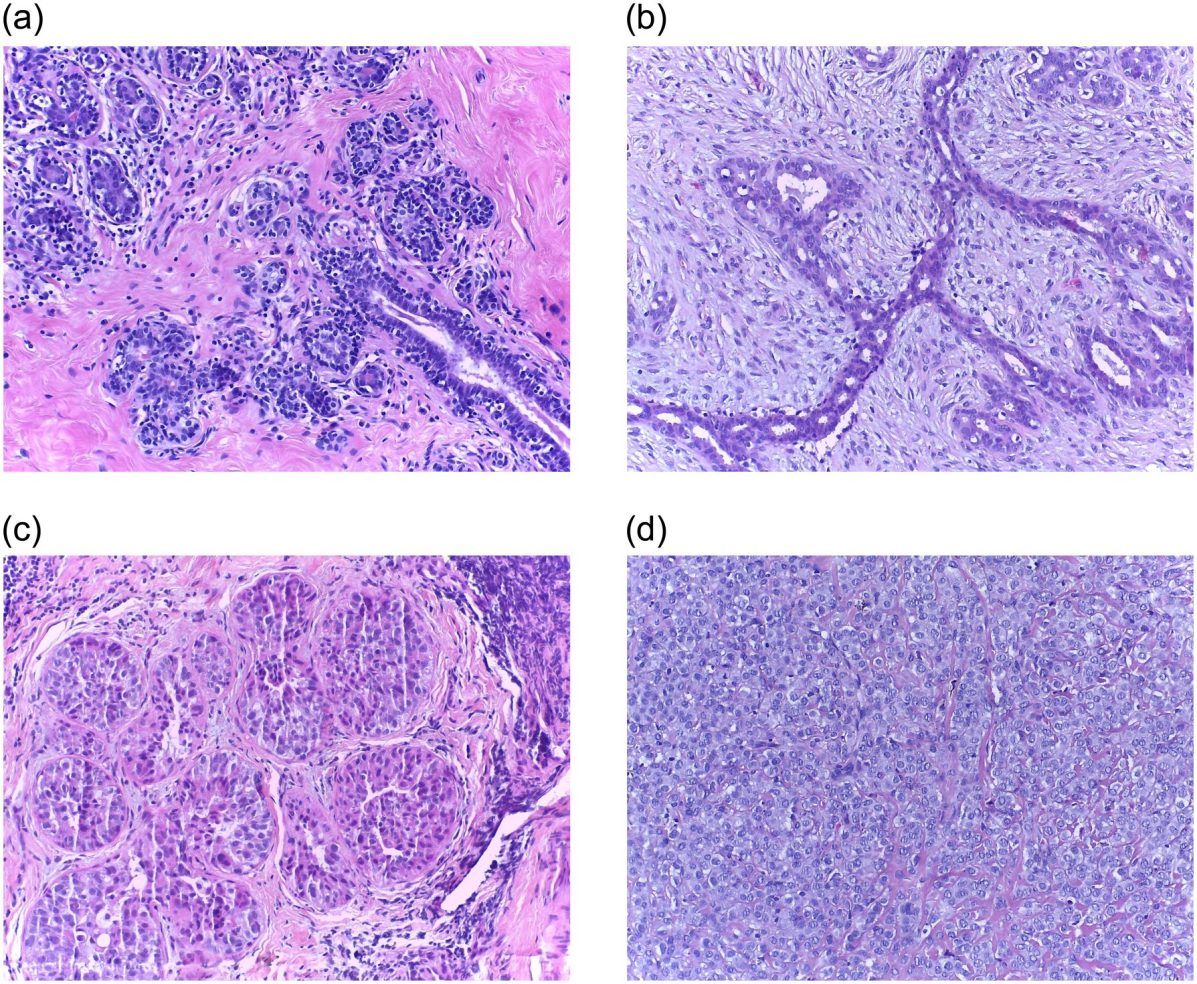
Examples of Bioimaging 2015 dataset. (a) Normal; (b) Benign; (c) In situ; (d) Invasive.

**Fig 8:**
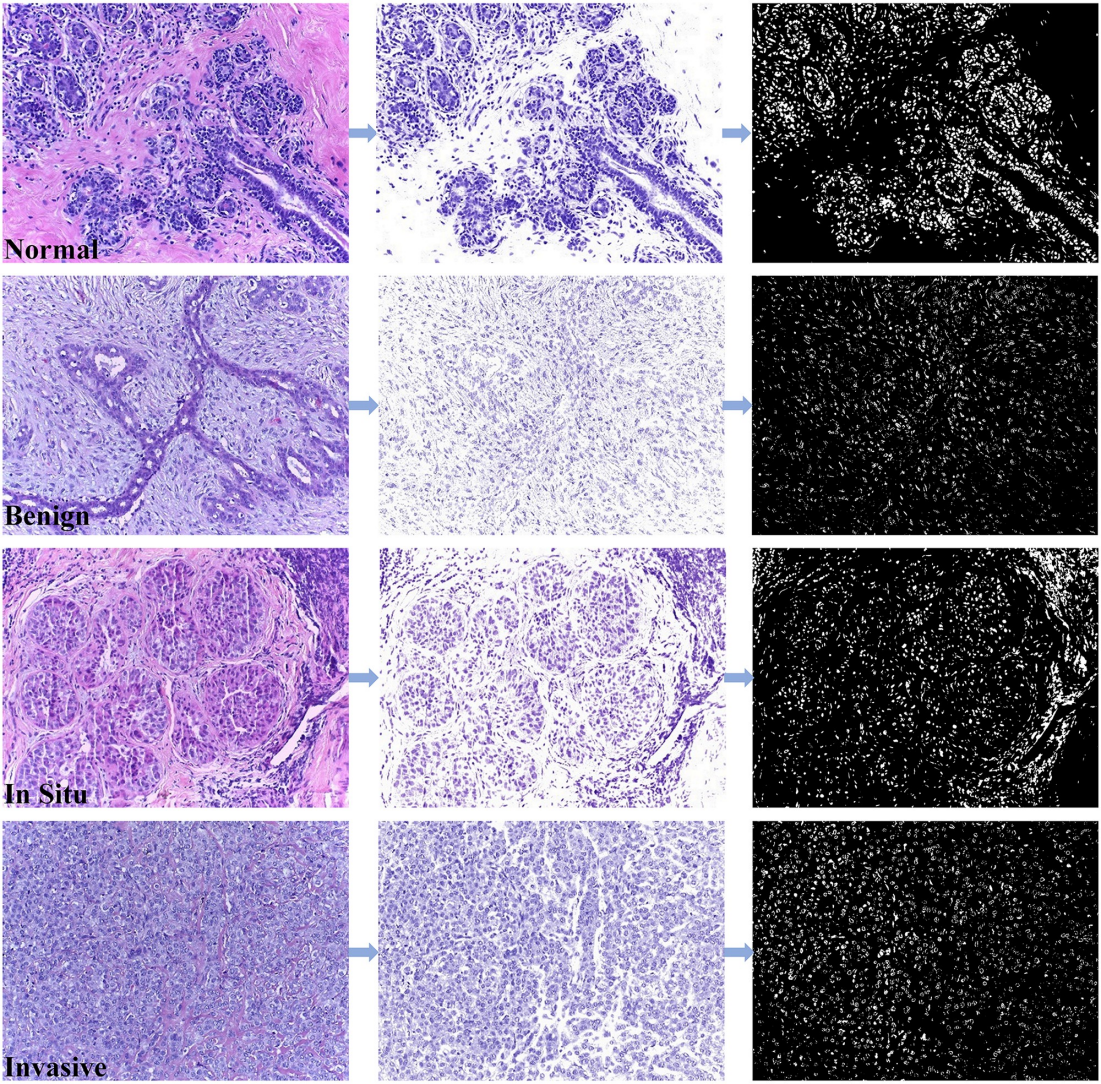
Segmentation results of sample images in bioimaging 2015 dataset based on two-stage nuclei segmentation strategy. (a) the original image, (b) the stain separation image, and (c) the segmented image.

The breast cancer histopathological image data used in this paper is the Bioimaging Challenge 2015 Breast Histology Dataset [16]. All images in this dataset are digitized under the same acquisition conditions, with a magnification of 200× and a pixel size of 0.42 *μm* × 0.42 *μm* (2048 × 1536 pixels). The images are stained with Hematoxylin and Eosin (H&E). Due to the characteristics of hematoxylin and eosin, the protein in the histopathological images will be stained pink by eosin, and hematoxylin will stain the cell nuclei blue-purple. All images are divided into four categories: normal, benign, in situ and invasive. Normal and benign tissues can be categories as non-carcinoma, and in situ carcinoma and invasive carcinoma can be categories as carcinoma, as shown in Fig 7. The images were labeled by two experienced pathologists, and the images with disagreements between the pathologists were discarded. The dataset consists of a training set of 249 images and a test set of 36 images (where 16 images have the increased ambiguity, called the extended test data). Table 1 shows the distribution of the dataset. Fig 8 shows the segmentation results of the proposed segmentation method for the complete image.

**Table 1:** Distribution of various histopathological images in Bioimaging 2015 dataset.

Category		Training data	Test data	
			Original test data	Extended test data
Non-carcinoma	Normal	55	5	4
	Benign	69	5	4
Carcinoma	In situ	63	5	4
	Invasive	62	5	4
Total		249	20	16

### 4.2 Experimental setup

In this paper, all the algorithms were performed under Matlab R2019a on a computer with a Windows 10 64-bit Professional platform and 8 GB RAM.

A series of pre-processing on the breast cancer histopathological images in the Bioimaging 2015 dataset. The original images are scaled by 0.5 times to obtain the images with a size of 1024 × 768. Then, 20 image patches are randomly cropped with a size of 512 × 512 from each image after scaling. If the number of cropped image patches is too small, it is difficult to ensure that the patches contain complete image information, and if the number of cropped image patches is too large, it may contain redundant information, so we choose to crop 20 image patches, which ensures that the patches can contain enough information and avoid redundant information. These two steps not only preserve the effective information of the original images, but also augments the dataset reasonably. And random cropping the images reduces the contingency of the experimental results.

The SVM with radial basis kernel function is used to be the classifier to make the tumors classified into non-carcinoma and carcinoma, where the penalty parameter *c* is 2 and the kernel function parameter *g* is 1. The image patches and the whole image are studied separately in the experiments. The image labels are obtained by majority voting, that is, for each test image, if more than 10 image patches are classified to be non-carcinoma, the image is classified to be non-carcinoma, otherwise it is classified to be carcinoma. In addition to the classification accuracy, the sensitivity, specificity, precision and F1_score are also taken to be the metrics of evaluating the classification performance for patch-wise and image-wise. The sensitivity represents the probability that carcinoma samples are correctly diagnosed in all carcinoma samples, the specificity represents the probability that non-carcinoma samples are correctly diagnosed in all non-carcinoma samples, and the precision represents the probability of correctly diagnosed carcinoma samples in samples that are diagnosed as carcinoma, and F1_score is the harmonic average of the sensitivity and the accuracy, which it is used to measure the balance of the two metrics. The formulas of the evaluation metrics are as follows [44].

$$Acc=\frac{TP+TN}{TP+FP+TN+FN},$$ (7)

$$Se=\frac{TP}{TP+FN},$$ (8)

$$Sp=\frac{TN}{TN+FP},$$ (9)

$$Pr=\frac{TP}{TP+FP},$$ (10)

$$F1\_score=\frac{2\times TP}{2\times TP+FP+FN}.$$ (11)

where true positive (*TP*) represents the number of carcinoma samples classified as carcinoma, true negative (T*N*) represents the number of non-carcinoma samples classified as non-carcinoma, false positive (*FP*) represents the number of non-carcinoma samples incorrectly classified as carcinoma, and false negative (*FN*) represents the number of carcinoma samples mis-classified as non-carcinoma.

### 4.3 Experimental results

#### 4.3.1 Comparison of different color feature methods

To get the best color features of breast cancer histopathological image for classification, the color histogram, the color moment and the color auto-correlogram are used to extract the corresponding color features before and after stain separation, and the classification performances of different color features are compared. For convenience, color histogram is abbreviated as Color-Hist, color moment is abbreviated as Color-Mome, and color auto-correlogram is abbreviated as Color-Auto-Corr, the color features and their abbreviations are shown in Table 2. The comparable results of the patch-wise and the image-wise are shown in Tables 3 and 4.

**Table 2:** Color features and abbreviations.

Methods	Abbreviations
color histogram	Color-Hist
color moment	Color-Mome
color auto-correlogram	Color-Auto-Corr

**Table 3:** Comparison of different color feature methods at patch-wise.

Image type	Features	Accuracy	Sensitivity	Specificity	Precision	F1_score
Original images	Color-Hist	71.81%	87.50%	56.11%	66.60%	75.63%
Original images	Color-Mome	65.28%	81.39%	49.17%	61.55%	70.10%
Original images	Color-Auto-Corr	60.83%	51.67%	70.00%	63.27%	56.88%
Stain separation images	Color-Hist	66.53%	74.72%	58.33%	64.20%	69.06%
Stain separation images	Color-Mome	64.86%	83.61%	46.11%	60.81%	70.41%
Stain separation images	Color-Auto-Corr	**75.97%**	**68.33%**	**83.61%**	**80.66%**	**73.99%**

**Table 4:** Comparison of different color feature methods at image-wise.

Image type	Features	Accuracy	Sensitivity	Specificity	Precision	F1_score
Original images	Color-Hist	75.00%	88.89%	61.11%	69.57%	78.05%
Original images	Color-Mome	66.67%	83.33%	50.00%	62.50%	71.43%
Original images	Color-Auto-Corr	66.67%	61.11%	72.22%	68.75%	64.71%
Stain separation images	Color-Hist	63.89%	77.78%	50.00%	60.87%	68.29%
Stain separation images	Color-Mome	69.44%	83.33%	55.56%	65.22%	73.17%
Stain separation images	Color-Auto-Corr	**88.89%**	**77.78%**	**100.00%**	**100.00%**	**87.50%**

The experimental results from Tables 3 and 4 show that the color histogram features perform the best for breast cancer images without stain separation. However, color auto-correlogram features obtain the best performance after stain separation. From Tables 3 and 4, it is also observed that when the color auto-correlogram method is used to extract the color features of the breast cancer image obtained from stain separation, the classification accuracy, the sensitivity, the specificity and the precision and F1_score at the patch-wise are 75.97%, 68.33%, 83.61%, 80.66% and 73.99%, respectively, and those at the image-wise are 88.89%, 77.78%, 100%, 100% and 87.50%, respectively.

Therefore, the color auto-correlogram features after the stain separation are chosen to be fused with the CLBP texture features after nuclei segmentation, which are regarded to be the input of SVM for final classification of breast cancer histopathological images. It should be noted that the original images mentioned in this section all refer to image patches with a size of 512 × 512 obtained by random cropping, which are relative to the stain separated images and the nuclei segmentation images.

#### 4.3.2 Comparison of image segmentation results under different conditions

To verify the effectiveness of the two-stage nuclei segmentation strategy proposed in this paper for the classification of breast cancer histopathological images, the CLBP texture features are extracted from the original images indirectly, the images obtained by the watershed segmentation on the original images, and the nuclei segmentation images obtained by the two-stage nuclei segmentation strategy on the original images, respectively. The fused features indicate the fusion of the CLBP texture features and the color auto-correlogram features. The compared results of CLBP features and the fused features are shown in Tables 5 and 6 at the patch-wise and at the image-wise, respectively, where the watershed segmentation on the original images is abbreviated as watershed segmentation.

**Table 5:** Comparison of image segmentation results under different conditions at patch-wise.

Image type	Features	Accuracy	Sensitivity	Specificity	Precision	F1_score
Original images	CLBP	72.08%	60.28%	83.89%	78.91%	68.35%
Watershed segmentation	CLBP	73.33%	63.89%	82.78%	78.77%	70.55%
Two-stage nuclei segmentation strategy	CLBP	75.00%	74.44%	75.56%	75.28%	74.86%
Two-stage nuclei segmentation strategy	Fused features	**82.22%**	**72.22%**	**92.22%**	**90.28%**	**80.25%**

**Table 6:** Comparison of image segmentation results under different conditions at image-wise.

Image type	Features	Accuracy	Sensitivity	Specificity	Precision	F1_score
Original images	CLBP	75.00%	61.11%	88.89%	84.62%	70.97%
Watershed segmentation	CLBP	75.00%	61.11%	88.89%	84.62%	70.97%
Two-stage nuclei segmentation strategy	CLBP	77.78%	72.23%	83.33%	81.25%	76.48%
Two-stage nuclei segmentation strategy	Fused features	**91.67%**	**83.33%**	**100.00%**	**100.00%**	**90.91%**

From Tables 5 and 6, the experimental results show that the classification accuracy of the two-stage nuclei segmentation strategy proposed in this paper is better at the patch-wise and the image-wise. The fused features of CLBP features extracted from nuclei segmentation image obtained by the two-stage nuclei segmentation strategy and the color auto-correlogram features after stain separation perform better than the other image types. From Tables 5 and 6, we also observe that the classification accuracy, the sensitivity, the specificity and the precision and F1_score at the patch-wise are 82.22%, 72.22%, 92.22%, 90.28% and 80.25%, respectively, and those at the image-wise are 91.67%, 83.33%, 100%, 100% and F1_score is 90.91%, respectively.

#### 4.3.3 Comparison of different segmentation methods

To verify the validation of the two-stage nuclear segmentation strategy proposed for breast cancer histopathological images in this paper, the k-means clustering segmentation, Ostu threshold segmentation, minimum error threshold segmentation method and iterative threshold segmentation are employed to be compared on the Bioimaging 2015 dataset for performing the classifications of breast tumors to be non-carcinoma and carcinoma. For convenience, k-means clustering segmentation is abbreviated as k-means, Ostu threshold segmentation is abbreviated as Ostu, and minimum error threshold segmentation method is abbreviated as Min-Error, and iterative threshold segmentation is abbreviated as Iter, the segmentation methods and their abbreviations are shown in Table 7. All the comparable methods have the same experimental conditions. For every segmentation method, two kinds of different feature extractions are adopted to perform the classifications of the breast histopathological images, which are the corresponding classification experiments: the classification on the CLBP features extracted after the nuclei segmentation, and the classification on the fused features of CLBP features and color auto-correlogram features. Thus the experimental results are shown in Tables 8 and 9.

**Table 7:** Segmentation methods and abbreviation.

Methods	Abbreviations
k-means clustering segmentation	k-means
Ostu threshold segmentation	Ostu
iterative threshold segmentation	Iter
minimum error threshold segmentation	Min-Error

**Table 8:** Comparison of different segmentation methods at patch-wise.

Segmentation methods	Features	Accuracy	Sensitivity	Specificity	Precision	F1_score
k-means	CLBP	72.92%	77.22%	68.61%	71.10%	74.03%
Ostu	CLBP	68.75%	57.22%	80.28%	74.37%	64.68%
Min-Error	CLBP	66.39%	64.72%	68.06%	66.95%	65.82%
Iter	CLBP	67.92%	59.72%	76.11%	71.43%	65.05%
The proposed	CLBP	75.00%	74.44%	75.56%	75.28%	74.86%
k-means	Fused features	74.72%	64.44%	85.00%	81.12%	71.83%
Ostu	Fused features	71.39%	67.22%	75.56%	73.33%	70.14%
Min-Error	Fused features	68.61%	64.17%	73.06%	70.43%	67.15%
Iter	Fused features	70.28%	62.50%	78.06%	74.01%	67.77%
The proposed	Fused features	**82.22%**	**72.22%**	**92.22%**	**90.28%**	**80.25%**

**Table 9:** Comparison of different segmentation methods at image-wise.

Segmentation methods	Features	Accuracy	Sensitivity	Specificity	Precision	F1_score
k-means	CLBP	77.78%	66.67%	88.89%	85.71	75.00%
Ostu	CLBP	72.22%	61.11%	83.33%	78.57%	68.75%
Min-Error	CLBP	69.44%	66.67%	72.22%	70.59%	68.57%
Iter	CLBP	69.44%	61.11%	77.78%	73.33%	66.67%
The proposed	CLBP	77.78%	72.23%	83.33%	81.25%	76.48%
k-means	Fused features	83.33%	88.89%	77.78%	80.00%	84.21%
Ostu	Fused features	75.00%	72.22%	77.78%	76.47%	74.29%
Min-Error	Fused features	72.22%	72.22%	72.22%	72.22%	72.22%
Iter	Fused features	77.78%	72.22%	83.33%	81.25%	76.47%
The proposed	Fused features	**91.67%**	**83.33%**	**100.00%**	**100.00%**	**90.91%**

**Fig 9:**
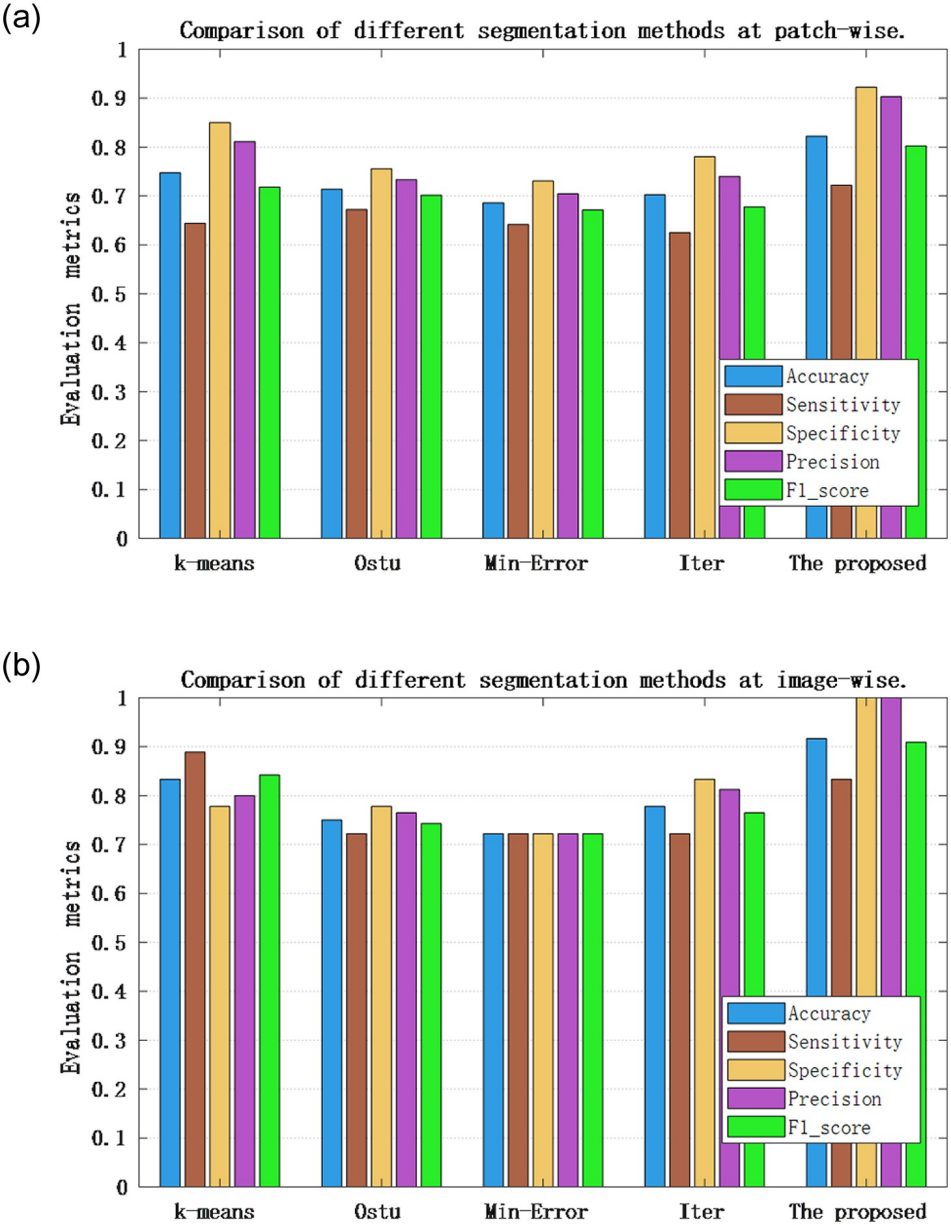
Comparison of the classification performance at the patch-wise and the image-wise on fused features. (a) Comparison of different segmentation methods at patch-wise, (b) Comparison of different segmentation methods at image-wise.

From Tables 8 and 9, it observed that the proposed two-stage nuclei segmentation strategy has obvious advantages over the other four compared segmentation methods both at the patch-wise and the image-wise and k-means clustering segmentation has better performance than the other three segmentation methods. It is worth noting that these segmentation methods have better classification results on fused features than those of CLBP features extracted from nuclei segmentation images. We also observe from Tables 8 and 9 that the classification accuracy, the sensitivity, the specificity and the precision and F1_score at the patch-wise are 82.22%, 72.22%, 92.22%, 90.28% and 80.25%, respectively, and those at the image-wise are 91.67%, 83.33%, 100%, 100%, and 90.91%, respectively. In particular, at the image-wise, the recognition rate is 91.67%, which indicates that 3 test images among 36 test images are incorrectly recognized, and the specificity is 100%, which indicates that all non-carcinoma images were correctly recognized, and all the 3 images are the samples of carcinoma category misclassified to be the non-carcinoma category. Fig 9 is the comparison of the classification performances at the patch-wise and the image-wise with the fused features.

**Fig 10:**
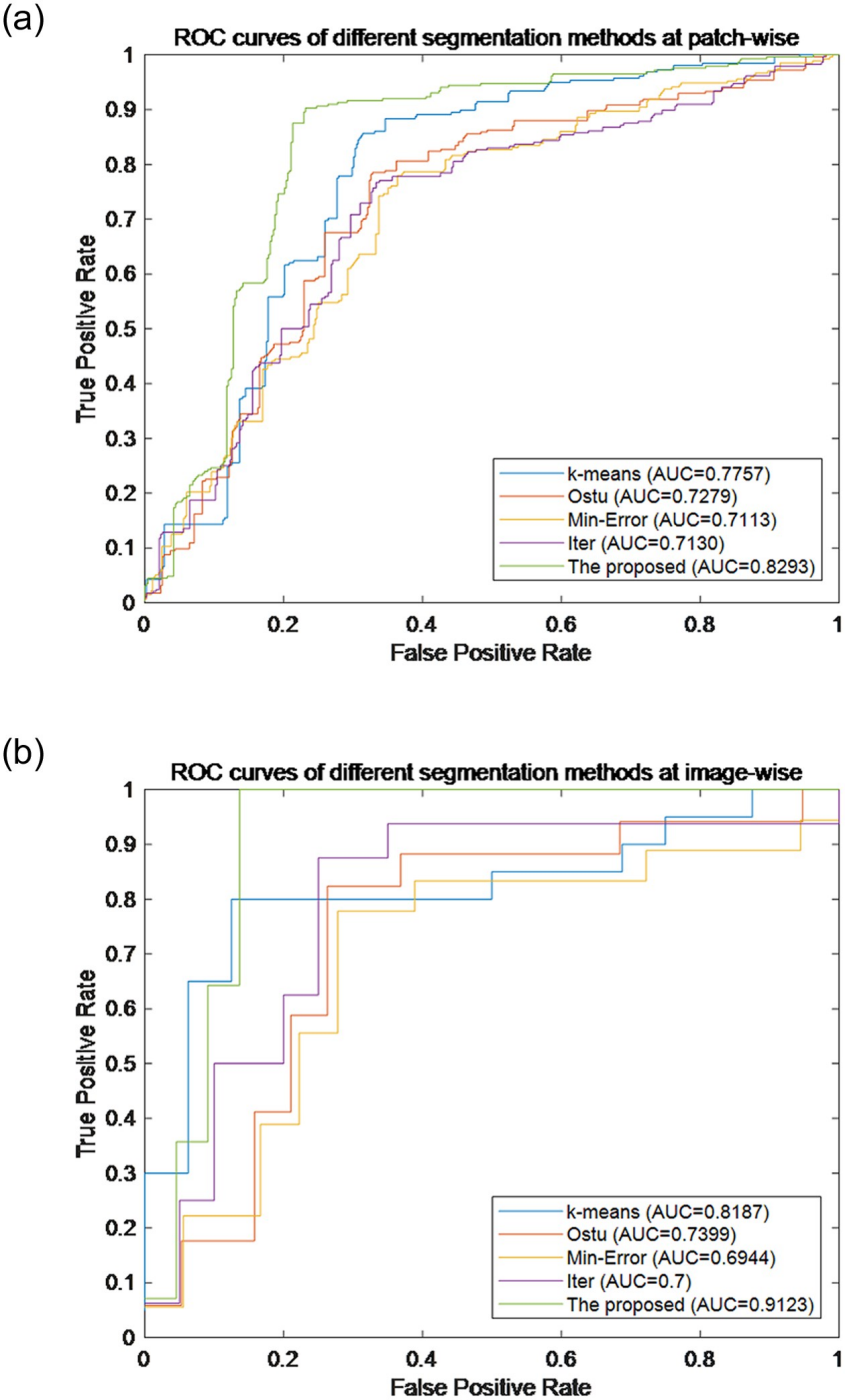
ROC curves of different segmentation methods. (a) ROC curves of different segmentation methods at patch-wise, (b) ROC curves of different segmentation methods at image-wise.

From Fig 9 we can see the advantages of the proposed method over other segmentation methods more clearly and intuitively. Therefore, the two-stage nuclei segmentation strategy proposed in this paper is superior to the other comparable segmentation methods. In order to compare the recognition performance of the proposed method with other segmentation methods more intuitively, the ROC curves and AUC values of different methods are compared, shown in Fig 10. From Fig 10, it can be seen that the proposed method significantly outperforms other methods in recognition performance whether it is patch-wise or image-wise.

#### 4.3.4 Results on the ICIAR 2018 challenge dataset

**Fig 11:**
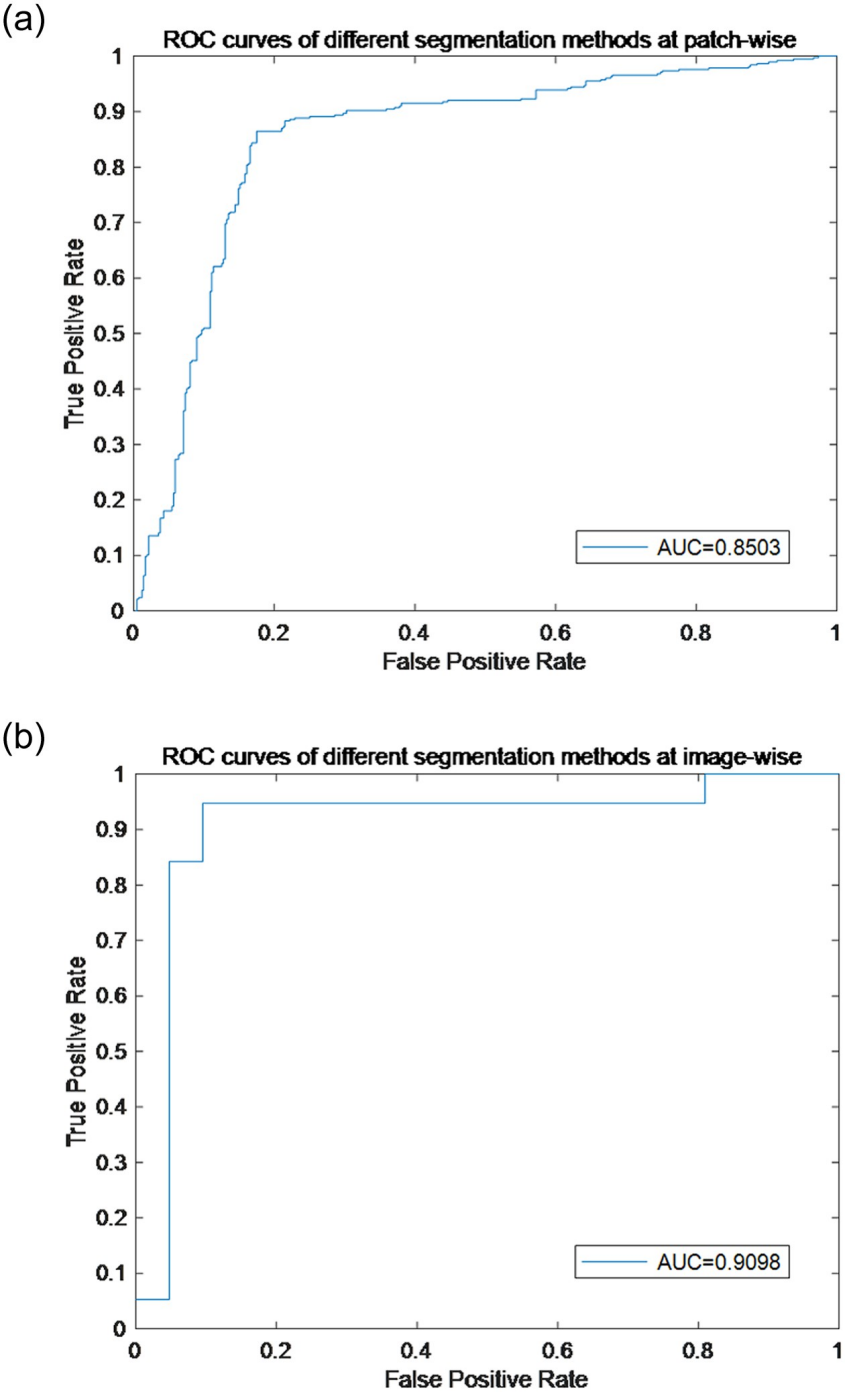
ROC curves of ICIAR 2018 challenge dataset. (a) ROC curves of the result at patch-wise, (b) ROC curves of the result at patch-wise.

We tested the proposed method on the ICIAR 2018 dataset, which is an extended version of the Bioimaging 2015 dataset, with the same image size and magnification as it [7]. ICIAR 2018 dataset consists of 400 breast histology images for training purpose and a separate hidden test set consisting of 100 images. We tested our method on this dataset by dividing the training set of this dataset, where we made 70% as training set, 20% as validation set and 10% as test set. And the classification accuracy, the sensitivity, the specificity and the precision and F1_score at the patch-wise are 84.38%, 81.50%, 87.25%, 86.47% and 83.91%, respectively, and those at the image-wise are 92.50%, 90.00%, 95.00%, 94.74%, and 92.31%, respectively. The results are shown in Table 10. This is the result of a competitive advantage over existing methods. The ROC curves and AUC values of the results are shown in Fig 11.

**Table 10:** Results on the ICIAR 2018 challenge dataset.

Image type	Accuracy	Sensitivity	Specificity	Precision	F1_score
Patch-wise	84.38%	81.50%	87.25%	86.47%	83.91%
Image-wise	92.50%	90.00%	95.00%	94.74%	92.31%

#### 4.3.5 Comparison of the current methods and the proposed method

To further verify the effectiveness of the two-stage nuclear segmentation strategy proposed in this paper, the classification accuracy of the proposed method in this paper and the current methods for breast cancer histopathological image classification at the image-wise are compared.

Table 11 shows the comparison of the classification performance of the proposed method in this paper and the existing methods on the Bioimaging 2015 dataset. It is observed from Table 11 that the proposed two-stage nuclei segmentation strategy method in this paper is significantly better than the methods in [16, 21, 23] on the same data set, but does not perform as well as the method in [24]. However, the related literatures are all using the deep learning algorithm, and the advantage of the deep learning algorithm is that it can get higher recognition accuracy, but the disadvantage is that a large number of labeled breast cancer histopathological images are required. Optimizing a large number of parameters also leads to a lot of time spent in the experiment. The method in this paper has good performance in realizing the recognition of carcinoma and non-carcinoma breast cancer histopathological images, and has the competitive ability in carcinoma and non-carcinoma recognition, can effectively replace the deep learning algorithm to a certain extent in breast cancer histopathology image recognition.

**Table 11:** Comparison of accuracy with previous methods.

Authors	Year	Dataset	Methods	Accuracy
Araújo et al. [16]	2017	Bioimaging 2015	CNN	80.6%
Araújo et al. [16]	2017	Bioimaging 2015	CNN+SVM	83.3%
Brancati et al. [21]	2018	Bioimaging 2015	ResNet	88.9%
Kassani et al. [23]	2019	Bioimaging 2015	VGG19, MobileNet, DenseNet	83.1%
Alom et al. [24]	2019	Bioimaging 2015	Inception Recurrent Residual CNN	99.05%
Roy et al. [20]	2019	ICIAR 2018	CNN	92.5%
Rakhlin et al. [22]	2018	ICIAR 2018	ResNet-50, VGG16	93.8%
Kassani et al. [23]	2019	ICIAR 2018	VGG19, MobileNet, DenseNet	95%
Our proposed method	-	ICIAR 2018	Two-stage nuclei segmentation strategy	**92.50%**
Our proposed method	-	Bioimaging 2015	Two-stage nuclei segmentation strategy	**91.67%**

## 5 Evaluation metrics of segmentation

In this paper, the Dice coefficient and Haus Dorff distance are used as evaluation metrics to measure the quality of the segmentation results. The Dice coefficient reflect more regional information and the Haus Dorff distance reflects more edge information. The calculation methods of the evaluation metrics are shown in formulas (12) and (13).

$$D=\frac{2\times \left|X\cap Y\right|}{\left|X\right|+\left|Y\right|}$$ (12)

where *D* is Dice coefficient, *X* is the prediction result and *Y* is Ground-truth.

H(X,Y)=max(h(X,Y),h(Y,X)) (13)

where *H* is Haus Dorff distance, $h(X,Y)=\underset{x\in X}{\text{max}}\left\{\underset{y\in Y}{\text{min}}\left\|x-y\right\|\right\}$, $h(Y,X)=\underset{y\in Y}{\text{max}}\left\{\underset{x\in X}{\text{min}}\left\|y-x\right\|\right\}$. Since the Bioimaging 2015 dataset is a classification challenge dataset, it mainly involves classification research and is not a dataset dedicated to segmentation, so Ground-truth is not included in the dataset. Therefore, we perform binarization processing under the same parameters for all images through threshold segmentation, try to approximate the obtained binary images as Ground-truth, and calculate the Dice coefficient and the Haus Dorff distance to evaluate the performance of the proposed segmentation method. When calculating the Dice coefficient, we average the Dice coefficients of all images, and take the maximum value among the Dice coefficients of each category.

As described in Section 4, k-means and our proposed method outperform the other comparable methods. Therefore, in this section, we take k-means and out proposed method to be compared by use of the Dice coefficient and the Haus Dorff distance. The results are shown in Table 12.

**Table 12:** Dice coefficient and Haus Dorff distance of segmentation results.

	Dice coefficient		Haus Dorff distance
	Average value	Maximum value	
k-means segmentation	0.4593	0.5961	9.9567
Our proposed segmentation	0.5884	0.7525	8.3606

The results show that the Dice coefficient of the proposed method is greater than that of the k-means cluster segmentation method, and the Haus Dorff distance is smaller than that of the k-means cluster segmentation method, which shows that the method proposed in this paper is superior to the k-means cluster segmentation method in terms of segmentation performance. But the value of the Dice coefficient is not very good, which may be caused by the fact that we do not have the real Ground-truth, but replace the Ground-truth with the binary image under the same parameter, and this approximate method of replacing the Ground-truth only It can be used as a reference to a certain extent, and cannot fully evaluate the segmentation performance.

## 6 Discussion and conclusion

The nuclei segmentation of histopathological images is of great significance for cancer diagnosis, grading and prognosis. The application of morphological standards in visual classification improves the accuracy of CAD systems and reduces human diagnosis errors. In this paper, a two-stage nuclei segmentation strategy, that is, a method of watershed segmentation based on histopathological images after stain separation, is proposed to make the dataset recognized to be the carcinoma and non-carcinoma recognition on the Bioimaging 2015 dataset. Compared with k-means clustering segmentation, Ostu threshold segmentation, minimum error threshold segmentation and iterative threshold segmentation, the proposed two-stage nuclei segmentation strategy performed the best and has the classification accuracy 91.67%, the sensitivity 83.33%, the specificity 100%, the accuracy rate 100% and F1_score 90.91%. In addition, compared with the current classification methods of breast cancer histopathological images, the proposed two-stage nuclei segmentation strategy method in this paper is also competitive and shows better classification performance. It is worth noting that those images with darker color and clearer imaging have better stain separation effect and better results of image segmentation. Therefore, our proposed method in this paper is affected by the image itself to a certain extent, such as the color depth and the clarity of the image.

In the future work, we will explore better nuclei detection and position methods to improve the effect of nuclear segmentation for histopathological images. And we will explore better feature extraction and fusion methods to further improve the classification performance of breast cancer histopathological images.
